# Complete Genome Sequence of Erwinia amylovora Strain TS3128, a Korean Strain Isolated in an Asian Pear Orchard in 2015

**DOI:** 10.1128/MRA.00694-21

**Published:** 2021-09-16

**Authors:** In-Jeong Kang, Duck Hwan Park, Young-Kee Lee, Sang-Wook Han, Youn-Sig Kwak, Chang-Sik Oh

**Affiliations:** a Division of Crop Cultivation and Environment Research, Department of Central Area Crop Science, National Institute of Crop Science, Suwon, Republic of Korea; b Applied Biology Program, Division of Bioresource Sciences, Kangwon National University, Chuncheon, Republic of Korea; c Crop Protection Division, National Institute of Agricultural Sciences, Rural Development Administration, Wanju, Republic of Korea; d Department of Plant Science and Technology, Chung-Ang University, Anseong, Republic of Korea; e Department of Plant Medicine, Institute of Agriculture and Life Science, Gyeongsang National University, Jinju, Republic of Korea; f Department of Horticultural Biotechnology, Kyung Hee University, Yongin, Republic of Korea; SIPBS, University of Strathclyde

## Abstract

Erwinia amylovora causes fire blight, a devastating disease of apples and pears. Here, we report the complete genome sequence and annotation of E. amylovora strain TS3128, which was isolated from Anseong, South Korea, where fire blight first occurred in 2015, using the PacBio RS II system.

## ANNOUNCEMENT

Erwinia amylovora causes fire blight, one of the most serious bacterial diseases of apple and pear trees. To understand the genomic characteristics of E. amylovora strains isolated in South Korea, complete genome sequencing of E. amylovora strain TS3128 was performed; this is a virulent strain that was isolated from an infected pear tree in Anseong, South Korea, in 2015 (1).

To isolate a single strain, infected pear leaf tissues were surface sterilized using 70% ethanol and were dissected into samples (5 by 5 mm). The dissected samples were macerated in 10 mM phosphate-buffered saline (PBS) (pH 7.2) (Biosesang, South Korea) in a sterile mortar. Single colonies observed after 48 h of incubation on King’s medium B at 27°C were isolated and subsequently purified. The purified strain was presumptively identified as E. amylovora by 16S rRNA and *recA* gene sequencing. Moreover, the primer pair AMSbL and AMSbR, based on the chromosomal *amsB* gene (2), was used to identify the strain as E. amylovora.

Total DNA was extracted from an overnight culture grown at 26°C in LB medium using the Wizard genomic DNA (gDNA) purification kit (Promega Corp., Madison, WI, USA). The integrity of gDNA was checked by agarose gel electrophoresis and quantified using a Qubit 2.0 fluorometer (Invitrogen, Carlsbad, CA, USA). The sequencing libraries were then prepared according to the manufacturer’s instructions for 20-kb template preparation using the BluePippin system (Sage Science, Beverly, MA, USA) with a DNA template preparation kit v1.0 (Pacific Biosciences [PacBio], Menlo Park, CA, USA). Briefly, 10 μg of gDNA was sheared to 20 kb using g-TUBEs (Covaris, Woburn, MA, USA), purified, and end repaired; blunt-end SMRTbell adapters were then ligated. The libraries were quantified using a Qubit 2.0 fluorometer (Invitrogen) and qualified using the DNA 12000 chip (Agilent Technologies, Waldbronn, Germany). The libraries were then sequenced using PacBio P6C4 chemistry in an 8-well SMRT Cell v3 in the PacBio RS II SMRT platform (3).

A total of 110,154 polymerase reads, with an average read length of 14,541 bp, were generated; the estimated genome coverage was 209-fold. Reads were polished using Pilon v1.22 (4) with default parameters implemented in Unicycler v0.4.7 (5). Circlator v1.4.0 was used to generate an ungapped circular form from the resulting contigs. Reads were assembled *de novo* using Hierarchical Genome Assembly Process (HGAP) v2 (PacBio) (6).

The complete genome of E. amylovora TS3128 comprises a circular chromosome of 3,804,041 bp and one plasmid of 28,288 bp, with average G+C contents of 53.6% and 50.2%, respectively. The chromosome and plasmid contain a total of 3,477 genes, including 3,237 protein-coding genes and 11 protein-coding genes, respectively, annotated with the NCBI Prokaryotic Genome Annotation Pipeline (PGAP) v4.11 (7). A total of 22 rRNA and 77 tRNA sequences were identified in the chromosome (Table 1). The plasmid in E. amylovora TS3128 shares 99% sequence identity (100% coverage) with pEA29 (8).

**TABLE 1:** Genome features of Erwinia amylovora strain TS3128

Feature	Data for:	
	Chromosome	Plasmid
Length (bp)	3,804,041	28,288
G+C content (%)	53.6	50.2
No. of coding sequences	3,237	11
No. of genes	3,448	29
No. of rRNAs	22	0
No. of tRNAs	77	0

### Data availability.

The genome sequence of E. amylovora strain TS3128 has been deposited in GenBank under accession numbers CP056034.1 (chromosome) and CP056035.1 (plasmid). Raw PacBio data have been deposited in the NCBI Sequence Read Archive (SRA) under the BioProject accession number PRJNA639906.

## References

[1] Myung IS, Lee JY, Yun MJ, Lee YH, Lee YK, Park DH, Oh CS. 2016. Fire blight of apple, caused by *Erwinia amylovora*, a new disease in Korea. Plant Dis 100:1774. doi:10.1094/PDIS-01-16-0024-PDN.

[2] Bereswill S, Bugert P, Bruchm Ller I, Geider K. 1995. Identification of the fire blight pathogen, *Erwinia amylovora*, by PCR assays with chromosomal DNA. Appl Environ Microbiol 61:2636–2642. doi:10.1128/aem.61.7.2636-2642.1995. 7618876 PMC167536

[3] Chin C-S, Alexander DH, Marks P, Klammer AA, Drake J, Heiner C, Clum A, Copeland A, Huddleston J, Eichler EE, Turner SW, Korlach J. 2013. Non hybrid, finished microbial genome assemblies from long-read SMRT sequencing data. Nat Methods 10:563–569. doi:10.1038/nmeth.2474. 23644548

[4] Walker BJ, Abeel T, Shea T, Priest M, Abouelliel A, Sakthikumar S, Cuomo CA, Zeng Q, Wortman J, Young SK, Earl AM. 2014. Pilon: an integrated tool for comprehensive microbial variant detection and genome assembly improvement. PLoS One 9:e112963. doi:10.1371/journal.pone.0112963. 25409509 PMC4237348

[5] Wick RR, Judd LM, Gorrie CL, Holt KE. 2017. Unicycler: resolving bacterial genome assemblies from short and long sequencing reads. PLoS Comput Biol 13:e1005595. doi:10.1371/journal.pcbi.1005595. 28594827 PMC5481147

[6] Eid J, Fehr A, Gray J, Luong K, Lyle J, Otto G, Peluso P, Rank D, Baybayan P, Bettman B, Bibillo A, Bjornson K, Chaudhuri B, Christians F, Cicero R, Clark S, Dalal R, Dewinter A, Dixon J, Foquet M, Gaertner A, Hardenbol P, Heiner C, Hester K, Holden D, Kearns G, Kong X, Kuse R, Lacroix Y, Lin S, Lundquist P, Ma C, Marks P, Maxham M, Murphy D, Park I, Pham T, Phillips M, Roy J, Sebra R, Shen G, Sorenson J, Tomaney A, Travers K, Trulson M, Vieceli J, Wegener J, Wu D, Yang A, Zaccarin D, Zhao P, Zhong F, Korlach J, Turner S. 2009. Real-time DNA sequencing from single polymerase molecules. Science 323:133–138. doi:10.1126/science.1162986. 19023044

[7] Tatusova T, DiCuccio M, Badretdin A, Chetvernin V, Nawrocki EP, Zaslavsky L, Lomsadze A, Pruitt KD, Borodovsky M, Ostell J. 2016. NCBI Prokaryotic Genome Annotation Pipeline. Nucleic Acids Res 44:6614–6624. doi:10.1093/nar/gkw569. 27342282 PMC5001611

[8] Laurent J, Barny MA, Kotoujansky A, Dufriche P, Vanneste JL. 1989. Characterization of a ubiquitous plasmid in *Erwinia amylovora*. Mol Plant Microbe Interact 2:160–164. doi:10.1094/MPMI-2-160.

